# Post-Exercise Rehydration in Athletes: Effects of Sodium and Carbohydrate in Commercial Hydration Beverages

**DOI:** 10.3390/nu15224759

**Published:** 2023-11-12

**Authors:** Nhu Q. Ly, Karrie L. Hamstra-Wright, Craig A. Horswill

**Affiliations:** 1 Independent Researcher, Charlotte, NC 28278, USA; nhuqly@gmail.com; 2 Department of Kinesiology and Nutrition, University of Illinois Chicago, Chicago, IL 60608, USA; khamst1@uic.edu

**Keywords:** carbohydrate, dehydration, oral rehydration solution, sports drink, sodium

## Abstract

The effects of varying sodium (Na) and carbohydrate (CHO) in oral rehydration solutions (ORS) and sports drinks (SD) for rehydration following exercise are unclear. We compared an ORS and SD for the percent of fluid retained (%FR) following exercise-induced dehydration and hypothesized a more complete rehydration for the ORS (45 mmol Na/L and 2.5% CHO) and that the %FR for the ORS and SD (18 mmol Na/L and 6% CHO) would exceed the water placebo (W). A placebo-controlled, randomized, double-blind clinical trial was conducted. To induce 2.6% body mass loss (BML, *p* > 0.05 between treatments), 26 athletes performed three 90 min interval training sessions without drinking fluids. Post-exercise, participants replaced 100% of BML and were observed for 3.5 h for the %FR. Mean ± SD for the %FR at 3.5 h was 58.1 ± 12.6% (W), 73.9 ± 10.9% (SD), and 76.9 ± 8.0% (ORS). The %FR for the ORS and SD were similar and greater than the W (*p* < 0.05 ANOVA and Tukey HSD). Two-way ANOVA revealed a significant interaction with the ORS having greater suppression of urine production in the first 60 min vs. W (SD did not differ from W). By 3.5 h, the ORS and SD promoted greater rehydration than did W, but the pattern of rehydration early in recovery favored the ORS.

## 1. Introduction

Inadequate fluid intake during sports participation and training can lead to dehydration. Persistent dehydration at or beyond 2% of body mass negatively impacts performance during subsequent efforts and increases the risks of heat illness [1,2]. Prompt and adequate rehydration between training sessions and competitions is important particularly when the period to rehydrate is brief before returning to physical and environmental challenges [1,3].

Rehydration is a function of gastric emptying, intestinal fluid absorption, and retention of fluid to restore body fluid compartments. A comprehensive measure of these processes is the percentage of fluid retained after a defined recovery period following ingestion of a specified volume of fluid [4,5]. To restore euhydration after exercise, ingesting from 125 to 150% of the volume lost has been recommended to offset urine losses during the recovery preceding subsequent exercise [1,6]. In research protocols, the defined recovery is often 4–6 h long [5,7,8,9,10,11,12,13]; however, athletes often need to perform sooner than that. Additionally, ingesting substantial amounts of beverage can be discomforting and could impact performance when the calculated replacement volume is great [14]. Consequently, the composition of the rehydration beverage may play an especially important role when time is lacking for the rehydration process or ingestion of merely 100% of the sweat loss volume can be tolerated.

The ingredients in a sports drink that promote rehydration include sodium (Na) and carbohydrate (CHO) with sodium being the primary factor [1,5,7,8,15,16]. Sodium helps maintain blood osmolality, which suppresses renal excretion of water and promotes fluid retention [5]. Hydration beverages with Na concentration of at least 40 mmol/L have been shown to help restore Na balance in individuals who underwent exercise-induced dehydration [5,8]. Furthermore, beverages with 40 and 50 mmol/L Na promoted greater fluid retention than beverages containing 31 mmol/L or less (*p* < 0.05) and produced retention similar to that of a 100 mmol/L beverage [5,8]. In these studies, beverages were either devoid of any carbohydrates [5] or the carbohydrate was maintained at 2% for all Na levels [8]. To date, other electrolytes such as potassium have not been demonstrated to have a clear effect on retention [4,16].

Carbohydrate content might also promote fluid retention during rehydration by prolonging gastric emptying and intestinal absorption [1]. This effect is apparent when the beverage CHO content is high, in the range of 10 to 12% with a fixed sodium content [9,10]. Beverages containing 10% CHO, well beyond that in sports drinks, have been reported to reduce plasma volume initially, likely due to slower gastric emptying because of the high energy density and/or osmolality of the hypertonic solution drawing water into the intestinal lumen from the serosal space [9]. Comparing beverages with Na fixed at ~31–32 mmol/L, Evans et al. reported a 2% CHO beverage promoted greater plasma volume expansion within the first hour of recovery vs. water or 10% CHO, suggesting enhanced absorption [9]. Ultimately, though, the 2% CHO beverage did not differ from the water placebo for fluid retention at the end of the 6 h recovery. In contrast, the 10% CHO beverage outperformed water for fluid retention by the 6 h mark [9].

The effect of varying CHO content in rehydration beverages having 6% or less CHO such as in sports drinks and oral rehydration solutions (ORS) is equivocal. Osterberg et al. found no difference in post-exercise rehydration fluid retention (~75% of the ingested volume) for a 3% vs. 6% CHO [10]. Kamijo et al. reported that, during recovery after exercise, less urine was lost for a 6.5% CHO beverage vs. a 3.3% CHO beverage and water control, but the 3.3% beverage also had less fluid loss than the water trial [11]. The total percent retained was not reported, but the higher CHO beverage induced a greater fluid balance based on urine loss. To rehydrate, participants in both studies ingested 100% of their exercise-induced weight loss and, while the CHO varied, the Na was fixed at 18 mmol/L [10] or 21 mmol/L [11].

The question that remains is whether variations in CHO concentrations of between 2 and 6% enhance rehydration when Na content also varies in the beverage. A low-CHO, hypotonic beverage, i.e., ~2%, with a Na concentration above 40 mmol/L might promote equal or greater fluid retention compared to a sports drink with 6% CHO and only 10–20 mmol/L of Na. Dumke’s lab examined this and reported no difference in fluid retention for an ORS (3.4% CHO and 60.9 mmol Na/L) or sports drink (6% CHO, 18 mmol Na/L) [17]. However, the protocol involved minimal dehydration (~1.2% of body mass) and the ingestion of 150% of weight loss before exercise was completed, and it lacked a placebo or water-control trial as a frame of reference for the unique protocol [17]. The lack of studies on this question hinders decision-making by athletes and recommendations by sports nutritionists regarding beverage selection to optimize rehydration particularly as athletes chose to limit dietary CHO.

The purpose of the present study was to compare beverages that varied in both Na and CHO content within the range found in sports drinks for rehydration properties following exercise-induced dehydration in male athletes. The main outcome variable used to define completeness of rehydration was the percentage of fluid retained during a 3.5 h period following beverage ingestion. To explore the effect of inversely varying Na and CHO, two commercially available and commonly used rehydration beverages were administered in volumes that replaced 100% of the acute body weight loss. A water placebo was compared to an ORS containing 2.5% CHO and 45 mmol/L Na and a standard sports drink containing 6% CHO and 18 mmol/L Na. We hypothesized that the higher Na, lower CHO beverage would promote the greatest rehydration.

## 2. Materials and Methods

### 2.1. Subjects

Physically fit males of ages from 18 to 30 y were recruited. Females were excluded to avoid the potential effects of estrogen fluctuations on water retention that might confound rehydration comparisons for the duration of testing a given subject [18]. The subject sample consisted of intercollegiate athletes, club sport athletes, several personal trainers, and several former military personnel, all of whom had to train regularly, i.e., >60 min a day at moderate to vigorous intensity, ≥3 d per week. All participants had to be free of any cardiovascular, metabolic, endocrine, or renal disease or dysfunction. Participants had to answer no to all seven questions on the PAR-Q, and each had to have a peak oxygen uptake (peak VO_2_) of ≥50 mL/kg/min. The study protocol was reviewed and approved by the institutional review board (no. 2013-0558), and written informed consent was obtained from each participant before testing. Physical characteristics are listed in Table 1.

Peak VO_2_ was measured using a metabolic cart (TrueOne 2400, Parvo Medics, Park City, UT, USA) during progressive resistance treadmill running. The running speed was 6–7 mph based on self-selection of the participant. After a two-minute warmup at 0% grade at the selected speed, the treadmill slope was increased by 1% every minute until volitional fatigue. An RER > 1.15, a heart rate within 10% of the age-predicted maximum, and a rating of perceived exertion ≥ 17 were used to confirm that maximum effort was delivered.

### 2.2. Experimental Design, Exercise Protocol, and Beverage Treatments

A randomized counter-balanced crossover design with double blinding was used. Each of the three trials occurred at least 3 days apart. During the 24 h period prior to each 8 h experiment, participants ate the exact same diet and did not exercise. To ensure a standardized diet with consistent energy and sodium intake, participants were provided with identical foods for meals during the 24 h period and surveyed for physical activity and diet to confirm consistency of conditions prior to each experiment.

**Figure 1:**
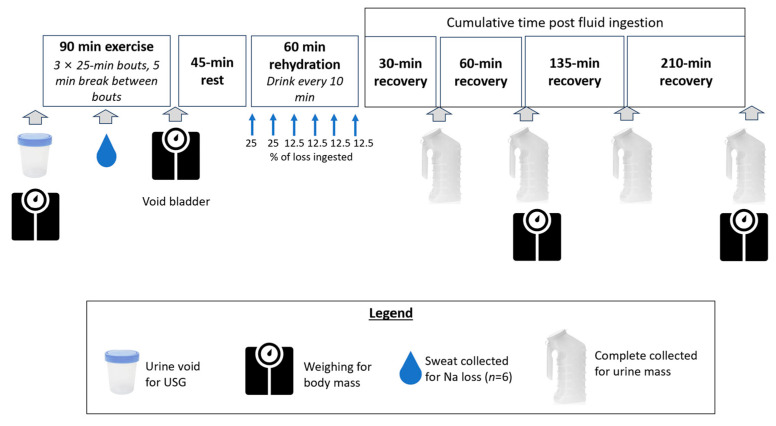
Sequence of the study protocol. Gray arrows indicate data collection times. Blue arrows during 60 min rehydration period indicate the percentage of volume ingested every 10 min.

A diagram of the protocol is presented in Figure 1.

To induce dehydration, participants exercised during a ~90 min session composed of three 25 min periods of intermittent-intensity exercise performed indoors after a 2 min warm-up. Exercise occurred on a treadmill, stationary bike, and elliptical machine; the order of use of the exercise machines varied between participants but was the same for an individual for all trials. Each 25 min period consisted of a fixed number of intervals at paces of walking (~3 mph), jogging (~7 mph), and running (10 mph), or the equivalent perceived intensities on the bike or elliptical machine (Table 2). Resistance settings for the bike and elliptical were identical for all three trials within a subject. A 5 min break was provided between periods for participants to dry off as needed, have body weight checked, and stretch.

During data collection on the final six participants, a pilot study was conducted to explore the role of sodium balance on the completeness of rehydration. Sweat samples were obtained during the second 25 min period of exercise, after sweating was well established, and analyzed for sodium concentration to estimate whole-body sodium loss during exercise. The collection technique has been used in prior rehydration studies [11] and is reliable but not without the potential to overestimate sweat sodium [19]. Just prior to resuming exercise, the forearm of each participant was sprayed thoroughly with distilled water to remove residual sodium on the skin and then thoroughly dried with a disposable, sodium-free towel. Immediately upon drying, a clear impermeable plastic bag was placed over the hand and forearm and secured with surgical tape to the inferior portion of the upper arm. At the end of that 25 min of exercise the bag was removed, and samples were analyzed immediately for sodium concentration.

During the exercise-dehydration period, no fluids were given to elicit a 2.5–3% reduction in body mass. Environmental conditions ranged from 22 to 29 °C (71 to 85 °F) and from 13 to 43% relative humidity. Following exercise completion, participants were weighed and rested for 45 min before consuming a volume of the beverage that replaced 100% of body mass lost. Beverages were ingested in six aliquots over a 1 h period given at the end of the trial. Specifically, 25% of the total volume was ingested every 10 min for the first 20 min; thereafter, 12.5% of the volume was ingested at four 10 min intervals.

The composition of the beverages is presented in Table 3.

Each participant ingested a water placebo (with flavored powder, private label version of Crystal Lite^®^ (Signature Brand, Itasca, IL, USA), ORS (Pedialyte^®^, Columbus, OH, USA), or sports drink (Gatorade^®^, Chicago, IL, USA) during one of the three experimental sessions to replace 100% of the body mass lost and were compared for fluid retention. All beverages were grape-flavored, purple in color, and administered in opaque cups to prevent drawing attention to the beverage differences. Beverages, which were served at room temperature, were prepared according to the manufacturers’ specs by a colleague who was not involved in any of the data collection or beverage administration for blinding purposes.

### 2.3. Analyses and Computations

Retention of ingested fluid was determined by measuring the mass of urine excreted at complete voids at minutes 30, 60, 135, and 210 after beverage ingestion. Body mass was measured at the 60- and 210-min time points, after urine collection. Between measurements, other than when they walked to the rest room or scale for data collection, participants remained seated and watched movies, worked on computers, or read self-selected materials.

The amount of beverage provided to rehydrate participants and the amount of urine produced during the 3.5 h recovery period was weighed on a calibrated analytical balance that measured fluid mass to within 0.02 g (ICS439-SW digital scale, Mettler-Toledo, Toledo, OH, USA). Body mass was measured to within 0.001 kg using a calibrated industrial scale (ICS439-SW digital scale, Mettler-Toledo, Toledo, OH, USA). The coefficient of variation for triplicate measures for the range of pre-exercise body masses within a subject was 0.005–0.009%. The change in body mass at the various times of measurement indicated the extent of fluid loss and replacement needed for the subsequent rehydration. For all weight measurements, participants were measured in the nude behind a curtain with the digital screen positioned for the researcher to record. The initial body mass was used as the denominator when change over time was expressed as a percentage (%).

The cumulative amount of urine produced during recovery was determined as the sum of the masses collected at 30 min, 60 min, 135 min, and 210 min (3.5 h) after ingestion of the rehydration beverage. The percentage of fluid retained was the primary outcome variable that represented completeness of rehydration and was calculated as follows:

% retained = 100 × (total mass ingest − cumulative mass urine)/total mass ingested(1)

The specific gravity of urine was measured using a digital refractometer (ATAGO, Tokyo, Japan).

Sweat sodium concentration was measured using a handheld sodium analyzer (Horiba C-122, Kyoto, Japan) that was previously shown valid for such use [20] and confirmed for accuracy against standard solutions of sodium chloride in our lab. To determine the mass of sodium lost in sweat during exercise, the sodium concentration was multiplied by the change in body mass (assuming 1 kg mass lost = 1 L water lost). The amount of sodium ingested during rehydration after exercise was determined by multiplying the sodium content of the beverage (value listed on label or prior knowledge) by the amount of fluid ingested for that specific experiment. Sodium balance was determined by subtracting the estimated sweat sodium loss during exercise (in mmol units) from the sodium ingested after exercise (also in mmol).

### 2.4. Statistical Analyses

Means ± standard deviation (SD) were used to summarize the data. One-way ANOVA was used to compare pre-exercise body mass, amount (kg) of fluid loss, and % dehydration (change in body mass) and confirm each trial replicated the state and conditions of each participant for each experiment.

For the primary outcome variable, cumulative % fluid retained for the 3.5 h period, a 1-way ANOVA was used with the Tukey test applied for post hoc analysis. For variables assessed for change over time, a 2-way ANOVA adjusted for repeated measures was used. These variables included the volume of urine collected (fluid loss) at the standardized times and the change in body mass. When main effects and interactions were identified, a 2-way ANOVA was then applied for differences between time points and a 1-way ANOVA with Tukey tests was applied for data within a time point. An alpha level of 0.05 was selected for statistical significance.

For the sub-sample measured for sweat sodium loss, correlation and regression were used to determine the relationship between completeness of rehydration (% fluid retained over 3.5 h) and sodium balance (sodium ingested in the beverage minus sodium lost during exercise). For this, a sample size of 18 was obtained by combining data from six participants for the three treatments. While it can be argued that this violates the independence of data points for correlation, slight differences in sweat sodium concentration, variations in sweat rate and absolute sweat loss within a trial, and different dosages of sodium ingested due to beverage content and the volume needed to restore body mass contribute to the same participants being unique from himself for each of the three experiments.

## 3. Results

### 3.1. Consistency of Baseline State

No participants were withdrawn or dropped out of the study. One participant was excluded before the study for failing to have a VO_2_ max ≥ 50 mL/kg/min. Twenty participants were tested between August and December with ≤3 weeks passing between the three trials for a given participant. The final six participants participated between 22 March through 28 March, with their three trials completed in 7 days (3 days apart). We believe all participants maintained the same fitness level, pre-exercise hydration level, and degree of environmental acclimation for their three treatments. These characteristics were important to maintain for minimizing variability in sweat rate, volume, and sodium loss that otherwise could confound rehydration comparisons between trials. This was confirmed in part by the absence of statistical differences testing for an order effect (1-way ANOVA) with the mean values for initial body mass, % dehydration, and sweat rate observed for the treatments being nearly identical (*p* > 0.05, Table 4).

### 3.2. % Fluid Retained and Urine Excretion

**Figure 2:**
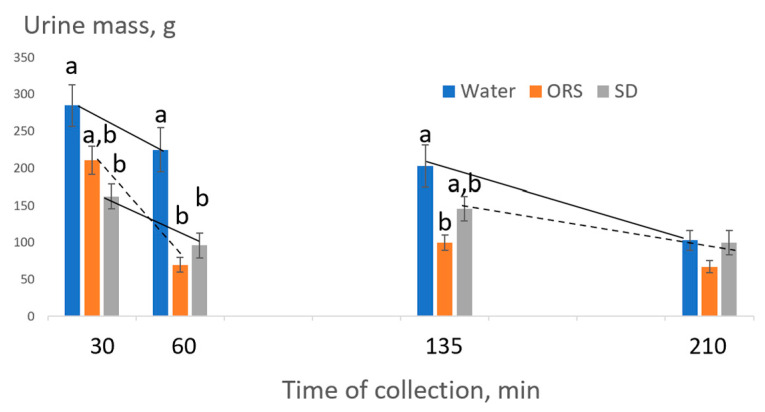
Mean urine production (grams) at collection times 30, 60, 135, and 210 min after ingestion of water (Wat), ORS, and sports drink (SD). Bars represent standard errors. Regardless of treatment, time points differed—less urine produced—at subsequent collections. Within a timepoint, different letters indicate beverage differences (*p* < 0.05 for post hoc tests of significant main effects for 2-way ANOVA). The dotted line indicates where significant interactions existed vs. solid lines for the rate of urine production (*p* < 0.05 for beverage-by-time interactions for 2-way ANOVA). Where lines are absent, no interactions occurred.

For the primary outcome variable, a difference was noted for cumulative % fluid retained by the ORS and sports drink compared to the water placebo at the end of the 3.5 h rehydration period (*p* < 0.05, Table 4). However, during the 3.5 h period, the pattern of retention differed among the beverages. Figure 2 presents the urine amount at each of the collection points following beverage consumption.

The 2-way ANOVA indicated a significant main effect for time and a time-by-treatment interaction (*p* < 0.05). Subsequent analyses were completed for treatment differences within a time point and the interactions (i.e., slopes of the lines) for sequential time periods of urine mass excretion. At the 30 min collection, significantly more urine was excreted for the water placebo than for the sports drink trial. At 60 min, the water placebo promoted more fluid loss than the sports drink or ORS and by the 135 min collection, the water placebo stimulated more fluid loss than ORS. For interactions between time points, akin to urine excretion rate, a significant interaction was found between the 30 min and 60 min collections for sports drink vs. ORS, indicating a greater rate of suppression of urine production by ORS. Similarly, a significant interaction was found for time points 135 min and 210 min for the sports drink vs. water, suggesting a greater decrease in urine excretion rate for the water placebo presumably because of so much fluid already having been excreted.

### 3.3. Body Mass

**Figure 3:**
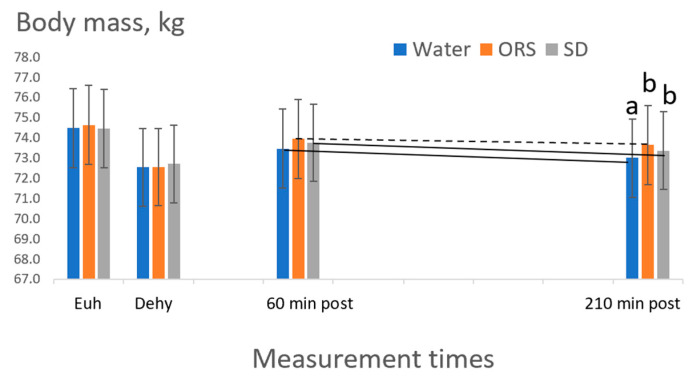
Mean body mass (kg) at each measurement point post ingestion of water (Wat), ORS, and sports drink (SD). Eu: euhydration before exercise (baseline); Dehy: dehydration measurement after 90 min of exercise. Bars represent standard errors. Regardless of treatment, body mass differed at Dehy from other times and body mass was lower at 60 min and 210 min vs. Eu. Within a timepoint, different letters indicate beverage differences (*p* < 0.05 for post hoc tests of significant main effects for 2-way ANOVA). The dotted line indicates where significant interactions exist vs. solid lines (*p* < 0.05 for beverage-by-time interactions for 2-way ANOVA).

Figure 3 presents the means for body mass at key time points of the study and compares the effects of the three beverage treatments.

A 2-way ANOVA indicated a main effect for time and for the time-by-treatment interaction (*p* < 0.05). Body mass was significantly reduced after exercise compared to pre-exercise (*p* < 0.05). Within any time point, no statistical differences were found between treatments for the absolute body mass (kg). However, for the interaction, a significant difference in the pattern (slope) for body-mass change appeared between the 60 min measurement and the final measurement at 210 min (Figure 3), such that the decline in body mass during the ORS trial was lesser than the decline for the other two beverages. This suggests the potential for higher sodium to be beneficial in maintaining hydration, compared to greater body mass decline for beverages with less or no sodium.

### 3.4. Sodium Balance

Table 5 summarizes the mean values for sodium lost, sodium ingested, sodium balance, and fluid retained for the pilot study on sodium balance and fluid retention.

**Figure 4:**
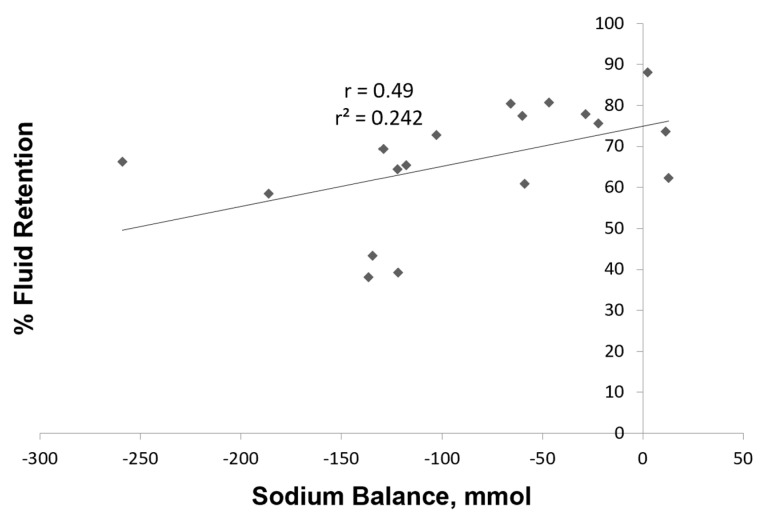
Relationship between fluid retained and sodium balance for six participants in the three trials (18 data points represented by diamonds). The r value was 0.49 (*p* < 0.05).

While large variability exists in sweat sodium loss between participants, the coefficient of variation for the three mean values of sweat sodium was ~10% to demonstrate consistency across trials. A negative value indicates an imbalance or deficit, which occurred in most cases for our participants. Converting mmol to mass units, the average losses ranged from 2.6 to 3.2 g during the 90 min workout sessions. Figure 4 depicts the relationship between sodium balance and % fluid retained. The relationship was statistically significant with r = 0.49 (*p* < 0.05), suggesting that reducing the sodium imbalance promotes greater rehydration by the end of the observation period.

## 4. Discussion

The primary hypothesis, that the beverage with the higher Na content and lower CHO would promote greater completeness of rehydration than that of the beverage with lower Na and higher CHO, was not confirmed. Fluid retention did not differ for either the ORS or sports drink at the end of 3.5 h, but both promoted more complete rehydration than the water placebo. Comparing mean values, the ORS was 32% more effective and the sports drink was 27% more effective than the response to the water placebo. It was expected that Na might play the dominant role based on the literature supporting a Na-dose effect when CHO content was fixed [5,7,8]. However, it is possible that a CHO effect might offset the lower Na content within a beverage. The results from two prior studies were equivocal with one showing a CHO effect only for a 12% beverage, not 3% or 6% CHO [10], while the other supported a CHO effect on fluid retention that differed for 3.3% vs. 6.5% [11]. In both studies, the Na concentration was fixed at ~20 mmol/L. To our knowledge, this is the first study on rehydration following exercise-induced dehydration when the treatment beverages varied in Na and CHO inversely within the ranges typically found in ORS and sports drinks.

In contrast to the outcome after 3.5 h, the pattern of fluid retained within the recovery tends to support the ORS as promoting a more rapid recovery. The urine volume for the ORS was reduced significantly between 30 and 60 min (*p* < 0.05) and directionally lower at all subsequent collection times. The pattern suggests a play between absorption and retention characteristics. Theoretically, with rapid absorption, water alone moves quickly into the vascular space, reduces plasma osmolality, and expands the plasma water volume, both of which stimulate the renal system to excrete water [1,5]. Rapidly absorbed beverages that provide the osmolytes Na and glucose would keep plasma osmolality higher, reduce the stimulus for urine excretion, and result in more fluid retained [1,5,7]. A more slowly absorbed beverage with a higher carbohydrate beverage might prevent the sudden surge in PV that elicits kidney excretion of water at the expense of taking longer to restore body fluid compartments [1,9].

Our speculation is supported by prior research indicating beverage energy density [21,22,23] and/or tonicity [9,21,22] influence the rate of fluid uptake by the gut. The water placebo (0 Cal/L, ~0 mOsm/L) might be expected to empty from the stomach and be absorbed in the intestines faster than the energy-containing beverages, and it might be expected that the ORS (100 Cal/L, 260 mOsm/L) emptied and was absorbed faster than the sports drink (240 Cal/L, 335–350 mOsm/L). While Osterberg et al. reported no difference in the net fluid retained after ~4 h of recovery, they did observe an elevation in plasma volume 1 h post-ingestion of the 3% CHO beverage, unlike all other treatments including the water placebo and the 6% CHO treatment [10]. Research assessing gastric emptying rates with the nasogastric tube [22] and tracer appearance as an index of intestinal uptake rate [23] show absorption patterns consistent with our explanation of the change in urine excretion pattern during the rehydration period.

Sodium balance, not just the beverage Na content, is also a critical factor in the completeness of rehydration [5,13]. To explore this, we studied acute sodium balance during the trials for our final six participants. Mindful of the small sample size, we found no statistical difference in Na loss between treatments. Differences were found for Na balance, due to the amount contributed by each beverage, and the positive relationship between Na balance and % fluid retained supports the role of beverage sodium for completeness of rehydration. Our observations were consistent with prior work of others using different collection methods or estimations [5,8,12,13] and helped confirm the benefit of replacing more Na with a rehydration beverage regardless of beverage CHO content.

The magnitude of acute weight loss (~2.6%) was consistent with prior rehydration studies [4,5,7,8,9,10,11,12,14,15,16] even though the exercise protocol was unique by using varied intensities and brief breaks to mimic the efforts in team sports. At the end of 3.5 h of recovery, though, our participants did not achieve euhydration based on their initial body weight in part by protocol design for which participants ingested 100% of fluid replacement needs. Body mass averages were higher, however, with either CHO-electrolyte beverage vs. the water placebo (*p* < 0.05). Based on the difference of %BM restored from 100% (% dehydrated), athletes could have reported to a second training session or competition at 2% hypohydration after the water treatment, but they were only 1.3% and 1.5% dehydrated for the ORS and the sports drink, respectively (*p* < 0.05 vs. water). Dehydration of 2% is commonly regarded as the break point for diminished physical endurance and mental function primarily due to reduced plasma volume, sub-optimal cardiovascular capacity for exercise, and impaired thermoregulation [2,24,25,26]. Some evidence suggests that merely 1.5% dehydration diminishes physical performance [26].

## 5. Limitations

The study was limited by including males only in a controlled laboratory setting for an 8.5 h period using non-invasive methods (body weight and urine excretion), and it compared commercially available products to manipulate beverage Na and CHO concentrations. Participants were asked to not exercise during the 24 h period prior to each experiment and were interviewed to confirm this at the start of each trial. By standardizing their diet and physical activity prior to studies, we assumed but did not verify that for each trial, participants had similar muscle glycogen levels and insulin responsiveness, which could influence Na retention [11]. Nonetheless, we do not know whether carbohydrate balance or glycogen recovery might influence the completeness of rehydration.

Using the change in body mass to establish the change in hydration status has been criticized as inexact [27]. However, others have shown, theoretically [28] and empirically [29], that acute weight change is predominantly determined by sweat (body water) loss during exercise. Arguably, giving 100% of the mass change could over-prescribe the required beverage volume if the change in body mass was influenced by substrate oxidation. Because the protocols for exercise were identical for each subject during each trial and their initial state was standardized, the contribution of substrate oxidation to body mass change would be a systematic but consistent error between the trials and would not negate our conclusions [1].

The use of commercial products prevented isolated comparisons of single functional ingredients, and not all ingredients were compared. The ORS contained only glucose at 2.5%, which would favor osmolality and energy density for fast absorption, whereas the 6% CHO sports drink contained sucrose and glucose that upon hydrolysis would be 50:50 glucose:fructose and, hence, have a higher energy density and be hyperosmotic. The ORS also had three times the chloride and over six times the potassium compared to the sports drink. Chloride might be important for fluid retention given that it resides in the extracellular space with sodium, but its rehydration potential is unclear [1]. Potassium has not been shown to enhance fluid retention during post-exercise rehydration when sodium is adequate [4,12,16] and remains questionable regarding its contribution to intracellular rehydration [1]. Despite this, the results help inform the coach and sports clinician toward educated choices for hydrating athletes depending on the time limit for completing the rehydration process. Additionally, the study results provide rehydration options depending on whether athletes desire or are required to account for sugar and calorie intake as a part of their recovery strategy between training sessions or competitions.

The methodology to quantify sweat Na losses had several limitations. A single-site collection may under-represent all losses, but the forearm site has been shown to correlate highly with whole-body loss [30]. Sweat collection occurred after the first exercise interval, so participants had likely achieved a steady-state sweat rate as required [19]. However, an impermeable covering like a patch or bag could stimulate higher sweat rates, elevate sweat Na concentration, and lead to an overestimate of whole-body Na loss compared to the true loss [30]. The mean sweat sodium values ranged from 60 to 72 mmol/L, which are high but not outside the range reported for athletes [31]. Any errors, though, would be systematic and still allow a reliable evaluation of the association between Na balance and degree of rehydration [18]. If our method led to an overestimate of sweat sodium loss, there would have been an even greater Na imbalance, which suggests more Na would be needed in the beverages to correct the imbalance and enhance the completeness of rehydration.

## 6. Conclusions

In summary, fluid retention during rehydration following exercise-induced dehydration of approximately 2.6% of body mass was superior in male athletes consuming 100% of fluid needs when beverages contained sodium and carbohydrate compared to drinking only water. Varying the CHO and Na within the typical range of sports drinks did not seem to vary for benefit by the end of the 3.5 h period. However, the ORS might be advantageous for earlier rehydration (within 2 h) based on a greater suppression of urine loss detected earlier in the recovery. The association between fluid retention and Na balance further confirms the role that beverage sodium content can play in rehydration.

## Figures and Tables

**Table 1:** Physical characteristics of the participants (*n* = 26).

Characteristic	Mean ± SD
Age, y	21.0 ± 3.0
Height, cm	177.8 ± 7.0
Weight, kg	74.5 ± 10.0
Body fat, %	13.1 ± 4.0
TM peak VO_2_, mL/kg/min	56.4 ± 6.9

Body fat was determined using skinfolds.

**Table 2:** Intermittent Variable-Intensity Exercise Protocol.

Phase	Interval No.	Intensity	Duration
Warm-up		“Jog”	2 min
Treadmill	1–5	Jog	1 min
		Walk	30 s
		Jog	40 s
		Sprint	20 s
		Jog	40 s
		Sprint	20 s
		Jog	40 s
		Sprint	20 s
		Walk	30 s
5 min break			
Stationary cycle	2–10	Jog	1 min
		Walk	30 s
		Jog	40 s
		Sprint	20 s
		Jog	40 s
		Sprint	20 s
		Jog	40 s
		Sprint	20 s
		Walk	30 s
5 min break			
Elliptical	11–15	Jog	1 min
		Walk	30 s
		Jog	40 s
		Sprint	20 s
		Jog	40 s
		Sprint	20 s
		Jog	40 s
		Sprint	20 s
		Walk	30 s

Order of machine use was the same within a participant for all 3 trials but varied between subjects.

**Table 3:** Composition of Beverage Treatments.

	Water Placebo	ORS	Sports Drink
Energy, Cal/L	~2.5 (CIT, MD)	100	240
Osmolality, mOsm/kg	0	270	330–380
CHO, g%	0	2.5% (GLU)	6.0% (SUC, GLU)
Sodium, mmol/L	0.33	45 (NaCl, Na-CIT)	18 (NaCl, Na-CIT)
Chloride, mmol/L	0	34 (NaCl)	11 (NaCl)
Potassium, mmol/L	0	20 (K-CIT)	3 (KH_2_PO_4_)
Zinc, mmol/L	0	0.12 (Zn-GLUC)	0

Nutrition form or source in parentheses. CIT: citrate; MD: maltodextrin; GLU: glucose; SUC: sucrose; Na: sodium; Cl: chloride; KH2PO4: potassium phosphate; GLUC: gluconate.

**Table 4:** Mean ± SD for % fluid retained, initial body mass, and % dehydration.

Treatment	Initial BM (kg)	% Dehy	% Fluid Ret *	% BM Restored *
Water Placebo	74.5 ± 10.0	2.57 ± 0.50	58.1 ± 12.6 ^a^	98.0 ± 0.9 ^a^
ORS	74.6 ± 10.1	2.57 ± 0.56	76.9 ± 8.0 ^b^	98.7 ± 0.3 ^b^
Sports Drink	74.5 ± 10.0	2.57 ± 0.52	73.9 ± 10.9 ^b^	98.5 ± 0.4 ^b^

*n* = 26. BM: body mass; Dehy: dehydration; Ret: retention. * ANOVA indicates statistical difference exists between treatments. Different superscripts indicate statistically significant differences using Tukey’s HSD (required difference: 7.125 for *p* < 0.05).

**Table 5:** Sodium (Na) flux (in mmol) for subset of six participants for the three treatments.

	Water Placebo	ORS	Sports Drink
Sweat Na lost	139.2 ± 64.1	113.0 ± 46.6	121.6 ± 71.0
Na ingested *	0 ± 0	89.4 ± 22.6	34.6 ± 9.8
Na balance *	−139.2 ± 64.1	−34.7 ± 55.6	−87.0 ± 62.7
% fluid retained ^	55.4 ± 17.6	72.5 ± 10.6	71.1 ± 7.9

* Treatment difference, *p* < 0.05 (1-way ANOVA). ^ Trend for treatment difference, *p* = 0.06.

## Data Availability

Data are contained within the article.
